# Is a delay in the introduction of human papillomavirus-based cervical screening affordable?

**DOI:** 10.1177/0969141318800355

**Published:** 2018-10-03

**Authors:** Alejandra Castañon, Matejka Rebolj, Peter Sasieni

**Affiliations:** 1 Centre for Cancer Prevention, Wolfson Institute of Preventive Medicine, Barts & The London School of Medicine and Dentistry, Queen Mary University of London, London, UK; 2 King’s College London, School of Cancer & Pharmaceutical Sciences, Cancer Prevention Group, Innovation Hub, Guys Cancer Centre, Guys Hospital, Great Maze Pond, London, UK

**Keywords:** Human papillomavirus screening, quality-adjusted life years, cervical cancer, screening implementation, implementation delays

## Abstract

**Objective:**

It often takes considerable time for sufficient evidence to accumulate to support implementation of new methods in routine screening. Where national screening programmes are already effective, switching to a more sensitive screening test may not be a priority. Although risk associated with overly rapid implementation exists, postponement is also associated with a (to date unquantified) missed opportunity to prevent deaths. This risk tends not to be addressed where effective screening methods are already in use. We here estimate the monetary value of a one-year delay in replacing cytology cervical screening with human papillomavirus testing.

**Methods:**

Using a previously validated model, we calculated the number of incident and fatal cervical cancers that would be diagnosed by 2030 in England, under the assumption that human papillomavirus testing replaces cytology in 2020 rather than 2019, and the monetary value of the quality-adjusted life years lost in preventable cases.

**Results:**

A one-year delay in the implementation of human papillomavirus screening would miss the opportunity to prevent 581 cases of cervical cancer, and lead to a loss of 1595 quality-adjusted life years (3.5% discount rate) with a monetary value of £32 million (at £20,000 per quality-adjusted life year).

**Conclusion:**

This is a measurable loss and should be considered in prioritising decision-making in screening.

## Introduction

In scientific circles, there is overwhelming support for replacing cytology with human papillomavirus (HPV) testing in primary cervical screening, and policy makers in the United Kingdom and elsewhere are planning for a rollout of HPV-based screening. HPV was first associated with cervical cancer in 1983,^1^ and nearly 20 years have passed since it was established as necessary for the development of cervical cancer.^2^ An algorithm for primary HPV screening with cytology triage was first published 15 years ago,^3^ and results from randomised trials^4–6^ showing that screening based on HPV testing prevents more cervical cancer than cytology screening^7^ were published almost 10 years ago.

The United States led the way in introducing HPV-based screening as an adjunct to cytology (also known as co-testing) in the mid-2000s. Countries where call/recall cervical screening programmes had yet to be established implemented HPV primary screening soon after, for example, Mexico from 2008^8^ and Turkey from 2014.^9^ By contrast, the pace in the European Union has been considerably slower, with HPV screening guidelines only being published in 2015.^10^ In the Netherlands, the Health Council announced its support for HPV-based screening in 2011 and the Ministry of Health made the decision to implement in 2013, but the full implementation was delayed from the initial plan in 2016 until early 2017. Denmark recommended HPV testing for primary screening in 2012 for women aged 60–65, but the test was not rolled out nationally until late 2014; in 2018, it was announced that the roll-out to women aged 30–59 will begin in 2019. Sweden made the decision to replace cytology with HPV testing for women aged 30 and above in 2015, with a gradual rollout starting in 2017. In Italy, HPV screening was recommended in 2013 and the rollout should be completed by 2018.^11^

In England, cervical screening is currently offered through a cytology-based call/recall programme three-yearly to women aged 25–49, and five-yearly to women aged 50–64. HPV testing is reserved for triage of equivocal cytology samples and follow-up after treatment of high-grade disease. In January 2016, the National Screening Committee recommended that HPV primary testing be adopted in the United Kingdom^12^ and the cancer outcomes strategy for England^13^ recommended national rollout by 2020. Six English sites have been piloting HPV primary screening since 2013 on approximately 13% of the screened population, with the intention of a future national rollout. Public Health England and the National Health Service (NHS), who are responsible for the screening programme in England, have until recently aimed to switch-over in April 2019,^14^ but are now working towards a December 2019 deadline.

Where national screening programmes are already extremely effective, switching to a more sensitive screening test may not be perceived as urgent. The cytology-based UK screening programme is highly effective in identifying and treating pre-invasive cervical lesions.^15^ Consequently, cervical cancer is rare among screened women, and it may be presumed that short delays in replacing cytology with HPV testing will have negligible consequences.

We estimated the excess number of screened women who will develop cervical cancer and the associated lost quality-adjusted life years (QALYs) under a scenario where the introduction of HPV screening is postponed by one year, while cytology-based screening continues.

## Methods

We estimated the excess in cervical cancers among women in England in a scenario where HPV primary screening is rolled-out nationally a year later than planned (in this example, in December 2020 instead of December 2019) (Table 1). We tackle this in two steps. First, we estimated cancer incidence to 2030 assuming cytology-based screening (three and five-yearly depending on age) and vaccination against HPV 16 and 18 is offered to cohorts born from 1990. We used data modelling for this.^17^ Separately, we estimated the proportion of cancers that would have been prevented by HPV testing by using data from a population-based case–control study.^15^ In both steps, we assumed that age-specific screening-coverage remains as in 2014/15 (Table 2).^18^

**Table 1.** Estimated number of excess cervical cancer cases in England by FIGO stage and age at diagnosis resulting from delaying replacing cytology with primary HPV screening for 12 months, and 5-year case fatality rates.

Age at diagnosis (years)	Estimated yearly number of cancers diagnosed with continued cytology screening^a^	Observed proportion of women with a negative cytology test prior to diagnosis^b^	Proportion of cancers prevented by HPV primary screening^c^	Total excess cancers	Stage 1A	Stage 1B	Stage 2	Stage 3+
				Excess number of cervical cancers^d^				
25–29	314.5	19.2	12.1	38.1	21.2	13.6	2.6	0.8
30–34	398.9	40.0	25.3	100.7	58.2	35.4	4.9	2.2
35–39	392.2	37.3	23.6	92.5	43.2	41.6	5.8	1.9
40–44	289.2	35.9	22.7	65.6	28.7	28.3	6.5	2.1
45–49	241.8	35.1	22.2	53.6	18.6	26.0	6.0	3.1
50–54	229.3	28.2	17.8	40.9	8.7	18.9	8.1	5.2
55–59	207.9	40.9	25.8	53.7	10.5	24.3	10.9	8.0
60–64	168.9	37.7	23.8	40.3	8.8	14.2	9.9	7.4
65–69	192.2	27.1	17.1	32.9	2.8	12.7	7.4	9.9
70–74	168.9	38.0	24.0	40.6	1.6	10.4	13.8	14.8
75–79	169.5	20.2	12.8	21.7	1.8	4.7	9.2	5.9
Total	2773.3			580.5	204.0	230.1	85.1	61.3
				5-Year case-fatality rates (%)^16^				
25.5–34				–	1.4	8.8	55.1	80
35–49				–	1.4	8.6	54.2	79.2
50–64				–	2.5	10.9	51.2	86
65–69				–	2.1	9.1	44.9	80.5
70–79				–	1.5	14.8	68.8	95.1

^a^For women aged 25 to 59, the observed cancers is an average of the estimated annual cervical cancers diagnosed between 2016–2020 and 2021–2025. For women aged 60–79, it is an average of the yearly number of cancers diagnosed from 2016 to 2030.

^b^Negative cytology test between 1.5 and 4.5 years prior to diagnosis at age 25–49; between 1.5 and 6.5 years at age 50–59 and between 1.5 and 11.5 years (at age 60 to 65) prior to diagnosis for those aged 60–79.

^c^We estimate the proportion prevented by HPV primary screening using the formula: Obs^d^0.632, where Obs: observed proportion with negative test.

**Table 2.** Screening coverage in England in 2014/15 by age group.

Age group	Cervical cancer screening coverage in 2014/15^a^		
	Regularly screened (%)	Lapsed (%)	Never (%)
25–29^b^	63.5	–	36.5
30–34	70.4	14.8	14.8
35–39	73.1	17.4	9.5
40–44	75.1	17.6	7.3
45–49	75.2	17.9	6.9
50–54	80.8	12.1	7.1
55–59	74.6	17.1	8.3
60–64	72.4	17.9	9.7

^a^Observed in 2014/15 cervical screening programme statistics,^18^ Table 3 (regularly screened defined as test within 3.5 years aged 25–49 and within 5.5 years aged 50–64).

^b^Since women are first invited for screening at age 25, women in this age group cannot be lapsed.

The first time the HPV test is offered to women as the primary screening test (i.e. the prevalence round), women will benefit if they are HPV positive but cytology negative; instead of receiving a three/five-year interval, they are re-called earlier and treated, if necessary. We used screening histories from the case–control study^15^ to estimate (by age group and FIGO stage) the proportion of women with a negative cytology test prior to diagnosis. We excluded cancers diagnosed within 18 months of a negative test because, for these cancers, it is probably too late to prevent the cancer by treating pre-invasive disease. However, we included cancers diagnosed up to and including 1.5 years after the next screen (because by screening now, we might be able to prevent those screen-detected on the next screen) (Table 1). Not all the cancers diagnosed following a negative test would have been preventable had the test been HPV rather than cytology. We have previously estimated^19^ that although 37.8% of cancers had a negative cytology in the appropriate window, only 23.9% (i.e. 63.2% of those with negative cytology) of additional cancers would be prevented by primary HPV testing. To take this into account, we multiplied the (age- and FIGO stage-specific) proportions of women with negative cytology by 0.632 (Table 1). Full methodological details can be found in online Appendix 1.

Age- and stage-specific five-year case fatality rates were taken from published literature.^16^ Ten-year cervical cancer relative survival^20^ is only slightly lower than the five-year survival, and so for fatal cases we assumed that, on average, women survive 2.5 years, and that survivors have the same remaining life expectancy as the general population.

A QALY is a generic measure of disease burden, including both the quality and the quantity of life lived. Institutions such as the National Institute for Health and Care Excellence (NICE) use this indicator of health benefit to compare various health interventions, and typically only recommend treatments if their cost per QALY is less than £20,000–£30,000. With this method, we estimated the monetary value of a timely HPV screening implementation, using England as an example. We did not attempt to quantify the risk associated with overly rapid implementation, but rather to calculate the benefits of early adoption.

The assumptions underlying the women’s life expectancy, the estimation of lost QALYs and its monetary value resulting from failing to prevent cervical cancer were all based on published parameters (Table 3). The total number of QALYs that would be saved with the switch to HPV-based screening in 2019 instead of 2020 was discounted to present value in 2019, to account for society’s tendency to prefer immediate benefits, rather than those accruing in the distant future. As recommended by NICE, we present results discounted with two interest rates 1.5% and 3.5%.^25^ Finally, the discounted QALYs were multiplied by NICE’s lower threshold incremental cost-effectiveness ratio, £20,000.^25^

**Table 3.** Parameters used in the estimation of the monetary value of lost QALYs associated with a one-year delay in the implementation of HPV-based cervical screening.

Parameter	Value		Source
Remaining life expectancy^a^	25––29 years	56.36 years	Office for National Statistics^21^
	30––34 years	51.46 years	
	35––39 years	46.59 years	
	40––44 years	41.77 years	
	45––49 years	37.02 years	
	50––54 years	32.35 years	
	55––59 years	27.81 years	
	60––64 years	23.42 years	
	65––69 years	19.20 years	
	70––74 years	15.22 years	
	75––79 years	11.58 years
Age–specific QALYs for the general population^b^	25––34 years	0.868	Janssen and Szende^22^
	35––44 years	0.864	
	45––54 years	0.824	
	55––64 years	0.803	
	65––74 years	0.766	
	≥75 years	0.742	
QALY detriments because of cervical cancer	Diagnosis, treatment	–0.285 for 0.116 years	Jit et al. ^23,24^
	Recovery	Linear change from –0.285 to –0.0305 in 1.5 years	
	Rest of life	–0.0305	
Threshold incremental cost–effectiveness ratio	£20,000		NICE^25^

Note: We used the following assumptions: (a) women were born on 1st July, (b) cervical cancers were diagnosed on 1st July when women reached the middle age of the respective age group, e.g. 27 for age group 25–29 years, (c) women with fatal cervical cancers died in on average 2.5 years after the diagnosis. These deaths represent the total mortality from cervical cancer.

^a^This is the average life expectancy that patients would have had they not developed a fatal cervical cancer. In our analysis, the remaining life expectancy for an age group was taken for year of age in the middle of the age group, e.g. at age 27 for age group 25–29.

^b^This is the average quality of life, on a scale from 0 to 1, that patients would experience throughout their life had they not developed cervical cancer. Women without cancer were assumed to have the same quality of life as the general population. These so-called population norms for English women show a decreasing average quality of life with increasing age, meaning that that as women age, there are progressively fewer QALYs that can be saved from preventing cervical cancer. Once a woman dies from the cancer, the QALYs that would have been experienced in absence of cancer are assumed to be lost.

QALY: quality-adjusted life years

## Results

At present, approximately 2500 cases of cervical cancer are diagnosed each year in England.^26^ We estimate that by 2030, 581 more cases could be prevented by introducing primary HPV testing in December 2019 rather than December 2020. Of these 581 cancers, 60% would have been diagnosed under age 50, and three-quarters at FIGO stage 1 (Table 1). Together, these 581 women would lose 1595 QALYs using a 3.5% discount rate (2285 using 1.5%). A comparison of this figure with other published estimates, and a discussion on how a change in screening interval could have an impact on the results is presented in online Appendix 2.

The monetary value of these QALYs (i.e. a saving with a timely implementation of HPV-based screening) is between £32 and 46 million, depending on discounting, over the women’s expected life spans. This means that, for every month the implementation is postponed, 48 additional women will be diagnosed with cervical cancer, at an estimated value of £2.7–3.8 million in terms of QALYs.

We have deliberately used conservative assumptions in our analysis. We assumed that all women who survive the first five years after cancer diagnosis will have normal life spans, and we have not taken into account the particularly severe QALY detriments during palliative care. The loss of quality of life among survivors was considered life-long, and therefore more affected by discounting, and we used the lower incremental cost-effectiveness threshold recommended by NICE.

Although these estimates are specific to England, they are informative for other countries. In fact, the opportunity cost of postponing HPV-based screening may be even greater in countries with less rigorous quality assurance and lower sensitivity of cytology than England.^27^ We have not attempted to estimate the cost to the health service of implementing change more rapidly. These and other considerations are discussed in online Appendix 3.

## Discussion

Making a change as profound as switching to a different screening test in a successful screening programme is no small task. The first challenge is obtaining official backing to implement a new screening test (or another health care policy). Once this is obtained, there is a risk that it could need to be reverted, causing reputational damage and sunk costs. In the case of HPV screening, this scenario is unlikely. The second challenge is preparing for the roll-out, during which aspects such as changes in laboratory organisation, contracting, staffing, quality assurance and, not least, revised management guidelines, all need to be considered, and this takes time. Reducing the time devoted to planning and preparing a roll-out to ensure earlier implementation could jeopardise the quality of the service, so realistic timescales and appropriate upfront investment are key to timely implementation of any new public health intervention. A multitude of factors can negatively affect the process. Evidence from organised screening programmes in Europe and elsewhere demonstrate that, even after the new policy has been agreed, a timely implementation thereafter is not guaranteed, and implementation delays experienced elsewhere can offer instructive examples.

Population-based call/recall databases underpin the running of organised screening programmes. Australia recently commissioned a single National Cancer Screening Register, with the objective of bringing together a number of existing databases. However, developing and implementing a screening registry solution, and the migration of existing databases, were more complex than expected, and this postponed the implementation date of HPV screening from May to December 2017. As a result, additional investment from the Government was needed to ensure continuation of cytology screening and staff retention.^28^ Similarly, the existing screening databases in England are unable to cope with the impending changes. In 2015 Capita, an FTSE 100 outsourcer began a £1bn contract to supply administrative support to NHS England. This contract included a redevelopment of the primary care support services (PCS) database, which handles several primary care services, including GP payments and screening call/recall. Capita’s original commitment was to introduce a standardised national screening database by June 2017.^29^ The complexities of the call/recall part of the PCS database were poorly specified, and there have been a number of complications. It is currently unclear when the database is expected to be ready for testing. Other countries could potentially avoid delays by considering computer systems that are purpose-built for screening.^30^ These could offer a platform that can more readily overcome the complexities of screening data, while still allowing a degree of individual tailoring.

It is often unpredictable external factors that derail the implementation process, even when it is reasonably well planned. The Netherlands was the first European country to announce its intention to implement HPV-based screening, with the process of change organised across several years and planned in detail.^31^ In 2015, however, this process was almost halted because of a media scandal due to incomplete disclosure of potential conflicts of interest of one of the country’s leading HPV researchers. In addition, the Netherlands opted for centralised procurement of a single HPV assay, and then faced lengthy legal battles with the unsuccessful competitors. Consequently, implementation was delayed until early 2017.

Announcing a profound change in policy can have unexpected consequences. In England, for example, uncertainty around laboratory configuration once HPV primary screening is implemented has begun affecting the cytology screening programme. Since the laboratories began reorganising in 2012 to support the use of HPV testing for triage of low-grade abnormal cytology and test of cure, the proportion of women who receive their screening test result within two weeks (one of the key performance indicators) has fallen from the target 98% in 2012/13 to 71.6% in 2016/17.^26^ This has been attributed to the increasing difficulty in maintaining staff numbers because cyto-screening is no longer an attractive or secure profession.

The benefits as well as the risks of more rapid implementation of innovations of proven efficacy should be formally evaluated at the beginning of the process. With such planning, countries could have allowed pilots of primary HPV testing for cervical screening to have been set up in 2007, with national rollout five years later, by 2012. In England, had rollout happened seven years earlier than it is now planned, by 2030 some 4000 fewer women would experience cervical cancer, leading to a QALY gain with a value of at least £224 million. In the case of HPV primary testing, this loss is even more troublesome, because screening can be done just as safely at longer intervals,^32^ which should be cost saving to the NHS. At present, screening programme and treatment costs amount to £157 million per year.^33^ It is expected that the cost of 6 and 10-yearly HPV-based screening would be about half that of 3 and 5-yearly cytology-based screening, leading to an additional direct saving of ∼£75 million per year (or ∼£500 million over seven years).

While careful planning is essential, sometimes there is a heavy price to pay for being overcautious.

## Supplemental Material

MSC800355 Supplemental material - Supplemental material for Is a delay in the introduction of human papillomavirus-based cervical screening affordable? Supplemental material, MSC800355 Supplemental material for Is a delay in the introduction of human papillomavirus-based cervical screening affordable? by Alejandra Castañon, Matejka Rebolj and Peter Sasieni in Journal of Medical Screening

## References

[1] Durst M Gissmann L Ikenberg H et al . A papillomavirus DNA from a cervical carcinoma and its prevalence in cancer biopsy samples from different geographic regions. Proc Natl Acad Sci U S A 1983; 80: 3812–3815. 6304740 10.1073/pnas.80.12.3812 PMC394142

[2] Walboomers JM Jacobs MV Manos MM et al . Human papillomavirus is a necessary cause of invasive cervical cancer worldwide. J Pathol 1999; 189: 12–19. 10451482 10.1002/(SICI)1096-9896(199909)189:1<12::AID-PATH431>3.0.CO;2-F

[3] Sasieni P Cuzick J. Could HPV testing become the sole primary cervical screening test? J Med Screen 2002; 9: 49–51. 2002/07/23. 12133918 10.1136/jms.9.2.49

[4] Naucler P Ryd W Tornberg S et al . Human papillomavirus and Papanicolaou tests to screen for cervical cancer. N Engl J Med 2007; 357: 1589–1597. 17942872 10.1056/NEJMoa073204

[5] Ronco G Giorgi-Rossi P Carozzi F et al . Results at recruitment from a randomized controlled trial comparing human papillomavirus testing alone with conventional cytology as the primary cervical cancer screening test. J Natl Cancer Inst 2008; 100: 492–501. 18364502 10.1093/jnci/djn065

[6] Kitchener HC Almonte M Gilham C et al . ARTISTIC: a randomised trial of human papillomavirus (HPV) testing in primary cervical screening. Health Technol Assess 2009; 13: 1–150. 10.3310/hta13510 19891902

[7] Ronco G Dillner J Elfstrom KM et al . Efficacy of HPV-based screening for prevention of invasive cervical cancer: follow-up of four European randomised controlled trials. Lancet 2014; 383: 524–532. 24192252 10.1016/S0140-6736(13)62218-7

[8] Panamerican Health Organization. *Integrating HPV testing in cervical cancer screening programs*: *a manual for program managers. Section 12: country experiences with implementing HPV test based screening programs*. Available at: https://www.paho.org/hq/dmdocuments/2016/manual-VPH-English-12.pdf (2016, accessed 6 September 2018).

[9] Gultekin M Zayifoglu Karaca M Kucukyildiz I et al . Initial results of population based cervical cancer screening program using HPV testing in one million Turkish women. Int J Cancer 2018; 142: 1952–1958. 29235108 10.1002/ijc.31212 PMC5888190

[10] European Commission. European guidelines for quality assurance in cervical cancer screening. Second edition – Supplements, http://bookshop.europa.eu/en/european-guidelines-for-quality-assurance-in-cervical-cancer-screening-pbEW0115451/?AllPersonalAuthorNames=true (accessed 21 October 2015).

[11] Eurogin Meeting. HPV induced cancers. Exploring knowledge, priorities and visions. In: *October 8–11* Amsterdam, 2017. Available at: https://www.eurogin.com/images/PDF/EUROGIN-2017.pdf (2017, accessed 6 September 2018).

[12] Public Health England (PHE). HPV primary screening in the cervical screening programme, https://phescreening.blog.gov.uk/2016/04/13/hpv-primary-screening-in-the-cervical-screening-programme/ (2016, accessed 15 June 2016).

[13] Report of the Independent Cancer Taskforce Achieving world-class cancer outcomes. A strategy for England 2015-2020, www.cancerresearchuk.org/sites/default/files/achieving_world-class_cancer_outcomes_-_a_strategy_for_england_2015-2020.pdf (2015, accessed 14 November 2017).

[14] Public Health England (PHE) and NHS England. Implementation of primary HPV testing in the English Cervical Screening Programme., www.britishcytology.org.uk/resources/Joint_PHE_and_NHSE_responses_to_questions_raised_by_the_BAC_and_IBMS.pdf (accessed 12 May 2017).

[15] Sasieni P Castanon A. NHSCSP Audit of invasive cervical cancer: national report 2009-2013, www.wolfson.qmul.ac.uk/centres/ccp/news/profiles/item/nhscsp-audit-of-invasive-cervical-cancer (2014, accessed 11 September 2017).

[16] Landy R Pesola F Castanon A et al . Impact of cervical screening on cervical cancer mortality: estimation using stage-specific results from a nested case–control study. Br J Cancer 2016; 115: 1140–1146. 27632376 10.1038/bjc.2016.290 PMC5117785

[17] Castanon A Landy R Pesola F et al . Prediction of cervical cancer incidence in England, UK, up to 2040, under four scenarios: a modelling study. Lancet Public Health 2018; 3: e34–e43. 29307386 10.1016/S2468-2667(17)30222-0 PMC5765529

[18] Health and Social Care Information Centre and Screening and Immunisations. Cervical Screening Programme, England – 2014–2015, www.hscic.gov.uk/catalogue/PUB18932 (2015, accessed 1 September 2017).

[19] Castanon A Landy R Sasieni P. By how much could screening by primary human papillomavirus testing reduce cervical cancer incidence in England? J Med Screen 2017; 24: 110–112. 27363972 10.1177/0969141316654197 PMC5490776

[20] Cancer Research UK. Cervical cancer survival statistics: one-, five- and ten-year survival for cervical cancer, www.cancerresearchuk.org/health-professional/cancer-statistics/statistics-by-cancer-type/cervical-cancer/survival#heading-Zero (2014, accessed 8 February 2016).

[21] Office for National Statistics National life tables, UK: 2013 – 2015, www.gov.uk/government/statistics/national-life-tables-uk-2013-2015 (2016, accessed 4 November 2017).

[22] Janssen B Szende A. Population Norms for the EQ-5D. In: Szende A Janssen B Cabases J (eds) Self-reported population health: an international perspective based on EQ-5D. Dordrecht: Springer Netherlands, 2014, pp.19–30. 29787044

[23] Jit M Brisson M Laprise JF et al . Comparison of two dose and three dose human papillomavirus vaccine schedules: cost effectiveness analysis based on transmission model. BMJ 2015; 350: g7584 25567037 10.1136/bmj.g7584 PMC4285892

[24] Jit M Chapman R Hughes O et al . Comparing bivalent and quadrivalent human papillomavirus vaccines: economic evaluation based on transmission model. BMJ 2011; 343: d5775. 21951758 10.1136/bmj.d5775 PMC3181234

[25] National Institute for Health and Care Excellence. Guide to the methods of technology appraisal. Available at: https://www.nice.org.uk/process/pmg9/chapter/the-reference-case (2013, assessed 6 September 2018). 27905712

[26] Screening and Immunisations Team. NHS Digital. Cervical Screening Programme, England – 2016–17, http://digital.nhs.uk/pubs/cervical1617 (2017, accessed 23 November 2017).

[27] Cuzick J Clavel C Petry KU et al . Overview of the European and North American studies on HPV testing in primary cervical cancer screening. Int J Cancer 2006; 119: 1095–1101. 16586444 10.1002/ijc.21955

[28] Australian National Audit Office. Procurement of the national cancer screening register. Australia: Author, 2017.

[29] NHS England. How NHS England is changing primary care support services, www.healthwatchbedfordborough.co.uk/sites/default/files/how_nhs_england_is_changing_primary_care_support_services_-_overview_for_patients_and_the_public.pdf (2016, accessed 4 November 2017).

[30] VCS Digital. canSCREEN™, www.vcsdigital.com.au/canscreen (2017, accessed 29 November 2017).

[31] van der Veen N Carpay MEM van Delden JA et al. Uitvoeringstoets wijziging bevolkingsonderzoek baarmoederhalskanker 2013 (RIVM rapport 225121002/2013) www.rivm.nl/Documenten_en_publicaties/Wetenschappelijk/Rapporten/2013/oktober/Uitvoeringstoets_wijziging_bevolkingsonderzoek_baarmoederhalskanker_2013. (2013, accessed 27 November 2017).

[32] Dijkstra MG van Zummeren M Rozendaal L et al . Safety of extending screening intervals beyond five years in cervical screening programmes with testing for high risk human papillomavirus: 14 year follow-up of population based randomised cohort in the Netherlands. BMJ 2016; 355: i4924. 27702796 10.1136/bmj.i4924

[33] Parliamentary Office of Science and Technology. Cervical Cancer, www.parliament.uk/documents/post/postpn316.pdf (2008, accessed 27 Nov 2017).

