# Supplementary material for: Is a delay in the introduction of human papillomavirus-based cervical screening affordable?
Source: J Med Screen. 2018 Oct 3;26(1):44–9. doi: 10.1177/0969141318800355 (PMC6376595; doi:10.1177/0969141318800355)
Supplement: MSC800355 Supplemental material - Supplemental material for Is a delay in the introduction of human papillomavirus-based cervical screening affordable? [file MSC800355_Supplemental_material.pdf]

Appendix 1. Methodological details

We estimated the average number of cancers diagnosed in England in 5-year age-groups (25-29, 30-34, etc) and calendar-bands (2016-2020, 2021-2025, and 2026-2030) assuming cytology screening continues. Full details of the model and its validation have been published previously.[A1] Briefly, we estimated incidence depending on screening and vaccination history and combined results from women with different histories. The model was built in four steps.

1. We use individual-level data (from a population based case-control study of cervical cancer diagnosed between 2007 and 2012)[A2] to obtain age-specific incidence rates in never-screened women. The numbers of cancers in the case-control study were weighted so that they matched, by calendar-year and age-group, cancer registration statistics between 2011-2015.
2. We estimated the relative risk for different birth cohorts from a modified age-period-cohort model.
3. Relative risks of cervical cancer by screening history were estimated using the case-control study.[A2] The relative-effect of HPV-based screening compared with cytology-based screening was taken from the literature.
4. The relative risk of HPV vaccination was estimated from a microsimulation model.[A3]

The model was calibrated to the average age-specific cervical cancer rates in England between 2011 and 2015. The estimated yearly number of cancers diagnosed with cytology screening are an average of cancers diagnosed between 2020 and 2024 for women <60yrs and between 2020 and 2030 for women aged 60-79 (the benefit of HPV testing in 2019 on cancer will be realised between 1.5 and 11.5 years after the test is taken). (Table 1)

To estimate the benefit of HPV testing relative to cytology, we use screening histories from the case control study[A2] to estimate (by age group and FIGO stage) the proportion of women with a negative cytology test prior to diagnosis. We exclude cancers diagnosed within 18 months of a negative test because, for these cancers, it is probably too late to prevent the cancer by treating preinvasive disease. However, we include cancers diagnosed up to and including 1.5 years after the next screen (because by screening now we might be able to prevent those screen-detected on the next screen).

For women with cervical cancer, we calculated the proportion with a negative cytology test between 1.5 and 4.5 years prior to diagnosis if aged 25 to 49, and between 1.5 and 6.5 years if aged 50 to 59. For women aged 60 to 79, we looked at the proportion of with a negative test (at age 60 to 65) between 1.5 and 11.5 years prior to their diagnosis (Table 1). Not all the cancers diagnosed following a negative test would have been preventable had the test been HPV rather than cytology. We have previously estimated[A4] that although 37.8% of cancers had a negative cytology in the appropriate window, only 23.9% (i.e. 63.2% of those with negative cytology) additional cancers would be prevented by primary HPV testing. The reduced efficacy is made up of: i) cancers that are both HPV and cytology negative; ii) cancers that will develop despite being HPV positive on screening; and iii) cancers that would have tested cytology positive will be HPV negative. To take this into account, we multiply the (age- and FIGO stage-specific) proportions of women with negative cytology by 0.632 (Table 1).

Appendix 2. Cancers prevented by HPV testing relative to cytology testing - comparison with published literature

Our group has previously estimated (in two separate publications) the reduction in cervical cancers with HPV primary testing relative to cytology. We use the same methodology in our papers but present slightly different results. Here we explain why they are different.

In the first manuscript published in 2017[A4] we estimated the reduction in cervical cancers at age 25 to 64 based on 2013 cancer incidence statistics, and report that they could be reduced by 487 (95% CI 394 to 563) cancers per calendar year or 3.4 (95% CI 2.8 to 4.0) per 100,000 women.

In the second manuscript published in 2018[A1] we estimated that in 2030 rates of cervical cancer in women aged 25-64 would be 12.4 per 100,000 if HPV screening is introduced soon, or 16.2 if we were to continue with cytology-based screening. With the ONS 2014 population projection for 2030 of 14.78 million women aged 25-64, that would amount to 562 fewer cancers annually aged 25-64.

In this manuscript we estimate the reduction in cancers at age 25 to 79 up to 2030, and hence estimate 19 cancers more (n=581) in this manuscript than in the 2018 one.

There remains uncertainty regarding screening intervals and triage test to be used once HPV testing is implemented in England. If the intervals are extended so that there are additional interval cancers, then the benefit of early adoption in terms of cancers prevented could be lessened.

Appendix 3. Other considerations

Vaccination

In England vaccination against HPV was introduced in 2008 for girls aged 12-13 as a free school-based programme, with catch-up for those aged 14-18. The youngest women receiving HPV primary testing in 2019 at age 25 will have been vaccinated against HPV 16/18 at the age of 14. Vaccination coverage at age 12 and 13 was 86%, at age 14 it was 69%, but only 40% at age 16 to 18.[A5] The potential effect of the nonavalent vaccine has not been considered here, as none of the women screened in 2019 or 2020 will have benefited. We have not accounted for the impact of herd immunity, but given how few women in the model would have benefited, its impact on test positivity rates and referral rates will be limited.

Cost to the health service

The aim of this analysis was to quantify, in a scenario where a high-quality screening programme exists, the monetary value (in terms of QALYs) of delaying the implementation of HPV primary screening owing either to a late decision or a late implementation but does not discriminate between the two.

An earlier start of HPV screening needs to consider the differences in prices between HPV and cytology testing. While HPV testing used to be considerably more expensive than cytology, recently published literature suggests that after the initial investment needed to process the HPV samples in the laboratory the running costs of an HPV based screening programme are similar if not cheaper to that of cytology (even without extending the screening interval).[A6,A7]

It must be considered that HPV testing is associated with a high proportion of false positive tests than cytology. In the future, the increase in false positive results can be offset by more specific HPV assays and if the screening interval is increased the initial cost of switching to

HPV testing and any ongoing costs associated with greater test positivity are likely to be rapidly recovered. Additionally, if the screening interval is extended, it could be that the cumulative risk of a false-positive test over 10 years (say) will be similar.

Appendix References

A1. Castanon A, Landy R, Pesola F, et al. Prediction of cervical cancer incidence in England, UK, up to 2040, under four scenarios: a modelling study. *Lancet Public Health* 2018; 3: e34-e43. 2018/01/09. DOI: 10.1016/S2468-2667(17)30222-0.

A2. Sasieni P and Castanon A. NHSCSP Audit of Invasive Cervical Cancer: National Report 2009-2013, http://www.wolfson.qmul.ac.uk/centres/ccp/news/profiles/item/nhscsp-audit-of-invasive-cervical-cancer (2014, accessed 11 September 2017).

A3. Landy R, Windridge P, Gillman MS, et al. What cervical screening is appropriate for women who have been vaccinated against high risk HPV? A simulation study. *Int J Cancer* 2017. DOI: 10.1002/ijc.31094.

A4. Castanon A, Landy R and Sasieni P. By how much could screening by primary human papillomavirus testing reduce cervical cancer incidence in England? *J Med Screen* 2017;24:110-112. DOI: 10.1177/0969141316654197.

A5. Sheridan A and White J. Annual HPV vaccine coverage in England in 2009/2010. , https://www.gov.uk/government/publications/annual-hpv-vaccine-coverage-in-england-in-2009-2010 (2010).

A6. de Kok IM, van Rosmalen J, Dillner J, et al. Primary screening for human papillomavirus compared with cytology screening for cervical cancer in European settings: cost effectiveness analysis based on a Dutch microsimulation model. *Bmj* 2012; 344: e670. 2012/03/07. DOI: 10.1136/bmj.e670.

A7. Lew JB, Simms KT, Smith MA, et al. Primary HPV testing versus cytology-based cervical screening in women in Australia vaccinated for HPV and unvaccinated: effectiveness and economic assessment for the National Cervical Screening Program. *Lancet Public Health* 2017; 2: e96-e107. 2017/12/19. DOI: 10.1016/S2468-2667(17)30007-5.
